# Community-Acquired, Extended-Spectrum β-Lactamase-Producing and Extensively Drug-Resistant *Escherichia coli* in a 28-Year-Old Pyelonephritis Patient Lacking Risk Factors

**DOI:** 10.3390/antibiotics10050533

**Published:** 2021-05-05

**Authors:** Connor W. Evins, Caroline M. Sutton, Sarah T. Withers, Jennifer T. Grier, Christine M. G. Schammel, Steven E. Fiester

**Affiliations:** 1 Department of Biomedical Sciences, University of South Carolina School of Medicine Greenville, Greenville, SC 29605, USA; cevins@email.sc.edu (C.W.E.); sarah.withers@prismahealth.org (S.T.W.); jgrier@greenvillemed.sc.edu (J.T.G.); 2 Department of Pharmacy, Prisma Health, Greenville, SC 29605, USA; caroline.sutton@unchealth.unc.edu; 3 Pathology Associates and Consultants, Greenville, SC 29605, USA; christine.schammel@prismahealth.org

**Keywords:** ESBL, *E. coli*, community-acquired, extensively drug-resistant, pyelonephritis

## Abstract

While *Escherichia coli* is a common cause of urinary tract infections and pyelonephritis, there are few documented cases of extended-spectrum β-lactamase (ESBL)-producing and extensively drug-resistant (XDR) isolates from the community resulting in infection requiring hospitalization, especially in individuals lacking risk factors. In the United States, exposure to ESBL-producing *E. coli* is typically nosocomial, whereas patients from developing countries often encounter ESBL-producing *E. coli* in the community through the consumption of contaminated food or water. Considering the rarity at which XDR *E. coli* isolates are encountered, there is also a scarcity of literature describing the successful treatment of ESBL-producing XDR *E. coli.* Here we present a case of an otherwise healthy 28-year-old female delicatessen worker infected with ESBL-producing and XDR *E. coli* without recent travel, antibiotic use, or healthcare contact, who required admission to the intensive care unit (ICU) with pyelonephritis and septic shock. Treatment with intravenous meropenem through a peripherally inserted central catheter (PICC) line at home was curative and follow up thereafter unremarkable. Given the patient’s lack of obvious exposure to and risk factors for an ESBL-producing XDR *E. coli* infection and the specific lack of risk factors for severe pyelonephritis requiring hospitalization, this case represents a unique addition to the literature and is of value to clinicians by describing successful treatment.

## 1. Introduction

Enterobacteriaceae are a large and diverse family of bacteria that infect humans in both the community and the healthcare setting. Some of these bacteria, most notably *Escherichia coli* and *Klebsiella pneumoniae*, are increasingly found to produce enzymes known as extended-spectrum β-lactamases, or ESBLs, which breakdown commonly used antibiotics such as cephalosporins and penicillin [1]. Some Enterobacteriaceae also carry other antibiotic resistance genes, including genes that inactivate fluroquinolones. ESBL-producing bacteria are resistant to most oral antibiotics, and patients often require intravenous carbapenem to combat infection. Since the 2000s, detection rates for ESBL-producing *E. coli* have been increasing. In 2017, 197,400 cases of ESBL-producing Enterobacteriaceae were identified in hospitalized patients, resulting in 9100 estimated deaths in the United States [2].

Several studies have suggested that antibiotic use can lead to the development of resistant bacteria and, subsequently, poor outcomes. Use of broad-spectrum antibiotics can independently increase the risk of subsequent ESBL-producing *E. coli* infections [3], and carbapenem resistant infections have occurred following short-term use of antibiotics for previously unrelated infections [4]. The development of multiple antibiotic resistances, especially in populations that commonly receive antibiotic treatments, poses a threat to patients in the healthcare setting. In fact, ESBL-producing *E. coli* has been shown to cause significantly more urinary tract infection (UTI) hospitalizations than non-ESBL-producing *E. coli*, with the ratio increasing in patients previously treated with antibiotic therapy or those with recent hospital admissions [5,6]. Additionally, patients who were administered carbapenem antibiotics, the most common antibiotic class used in treatment of ESBL-producing bacteria [7], and β-lactam β-lactamase inhibitor combination antibiotics [8] were noted to be at risk of increased mortality. In response to this, judicious use of antibiotics has been presented as a solution to mitigate the increase in frequency of UTIs caused by ESBL-producing bacteria [6].

The spread of ESBL-producing bacteria in the healthcare setting is typically thought to be through direct contact with contaminated surfaces or hands between healthcare workers or patients [2]. Recent travel to endemic areas has also been shown to spread ESBL-producing Enterobacteriaceae, in particular the strains that produce CTX-M enzymes [9]. A 2018 study showed that, of these ESBL-producing strains reported to the United States Centers for Disease Control and Prevention (CDC) from antibiotic susceptibility testing, the vast majority were found in patients that had traveled to South America within seven days of symptom onset [9]. Strains void of the resistance genes were more often associated with patients who did not report travel, thus the strains were determined to be acquired domestically [9]. This suggests that ESBL-producing strains may be more common outside of the United States and supports recent travel as a risk factor.

While *E. coli* is a common cause of urinary tract infections and pyelonephritis, specifically representing 85% of pyelonephritis cases [10], the risk of infection with a resistant isolate is infrequent in uncomplicated pyelonephritis and more common in complicated pyelonephritis (pyelonephritis due to a structural or functional abnormality in the urinary tract) [11]. Complicated pyelonephritis is characterized with a broader spectrum of signs and symptoms, greater chance of being caused by infection with a resistant bacterium and increased risk of complications such as abscess formation. Risk factors for uncomplicated pyelonephritis include increased sexual activity, frequent or recent history of UTIs, incontinence, and a history of UTIs in the patient’s mother [10]. However, for healthy community dwelling patients with few of these risk factors, development of uncomplicated pyelonephritis with a resistant bacterium requiring hospitalization is exceedingly rare [10]. Here we present a rare case of a young, immunocompetent, and otherwise healthy food services worker in the United States, with no functional or structural genitourinary abnormality, diagnosed with uncomplicated pyelonephritis as a result of an ESBL-producing and extensively drug-resistant (XDR) *E. coli* infection, without previous hospitalization, antibiotic treatment, travel, risk factors for uncomplicated pyelonephritis, or clear route of transmission and describe successful treatment of this infection.

## 2. Case Report

A 28-year-old Hispanic Spanish-only speaking female with no known past medical history and no known use of antibiotics presented to the emergency department with a one-week history of abdominal pain, nausea, vomiting, and subjective fever. The patient reported urinary frequency but denied dysuria. She reported that she had never experienced these symptoms before and denied history of UTIs. The patient also denied constipation, recent incontinence, diarrhea, vaginal discharge, headaches, chest pain, shortness of breath, cough, heart palpitations, vision changes, and fatigue. She had not had any recent sick contacts, recent travel, or long periods of immobility. The pain had steadily worsened and become localized to the left flank over the previous few days; however, the patient had not taken anything to help the pain or nausea. Family and sexual history were unremarkable. Vital signs revealed a heart rate of 117 beats per minute, temperature of 101.6 °F, and blood pressure of 83/44 mmHg. The abdominal exam was negative except for left CVA tenderness. A pelvic exam revealed no signs of cervicitis or pelvic inflammatory disease. A urine pregnancy test was negative. A computerized tomography scan of the patient’s abdomen was also negative for acute pathology. Urinalysis revealed cloudy urine, a small increase in leukocyte esterase, and an elevated white blood cell count of 12 (4 times upper limit normal). She was given normal saline (1 L), acetaminophen, and ceftriaxone with plans to discharge if vitals improved. Unresolved tachycardia (>100 bpm) and hypotension (77-109/39-84) resulted in admission to the intensive care unit with a diagnosis of pyelonephritis and septic shock.

Following admission, the patient remained hypotensive and tachycardic with worsening fatigue. A central line was placed, and following 5 L of normal saline, her vitals stabilized. A continuous normal saline drip was started (250 cc/hr). The patient continued to be febrile with fever reaching 105.1 °F (oral) despite acetaminophen, and she remained on ceftriaxone. On hospital day three, automated identification and antibiotic susceptibility testing from a urine specimen using the VITEK 2 (bioMérieux), according to the manufacturer’s instructions, confirmed the cause of infection as ESBL-positive, XDR *E. coli* (Table 1). Ceftriaxone was discontinued and replaced with meropenem every 8 h. On hospital day four, the patient’s vitals stabilized, and fever normalized; additionally, the flank pain, fatigue, and urinary frequency improved. She was discharged with a peripherally inserted central catheter (PICC) line on hospital day five. Meropenem was discontinued and the patient was transitioned to ertapenem at home for a total of 10-day carbapenem coverage. An infectious disease consult determined the patient had no contact with healthcare and no recent antibiotic use; however, she did work as a butcher handling raw meat in a supermarket. After further research into the patient’s place of employment, it was noted on the state food safety website that the meat market had several violations including soap not provided at all handwashing stations and the presence of pests. The documented violations were all surrounding the patient’s date of hospitalization; however, no swabs were collected at her place of employment and no tests performed to confirm the patient’s workplace as a possible route of transmission. She followed up as an outpatient with infectious disease six days after completing her 10-day course of carbapenem therapy for ESBL-producing XDR *E. coli*. She remained asymptomatic with no fever, dysuria, flank pain, nausea, vomiting, or fatigue. She did not report any side effects to the medication, or any pain, redness, or swelling around the PICC line. After completion of therapy, her PICC line was removed without complication or relapse in symptoms despite infection with this XDR isolate. Follow up was unremarkable, with no reoccurrence of UTI or urinary complaints to report.

## 3. Discussion

In the case presented herein, the patient was diagnosed with severe community-acquired pyelonephritis with no known source of infection. Her condition continued to deteriorate as the infection was not ameliorated with ceftriaxone, and she was admitted to the ICU with septic shock. Hospitalization for pyelonephritis in non-pregnant women less than 50 years old with no healthcare exposure is rather unique [10]. The unusually high morbidity in an otherwise healthy patient was explained when the causative agent of her UTI was identified and found to be not a community-acquired bacterial isolate with little resistance, but rather an XDR ESBL-producing *E. coli* resistant to nearly all antibiotics tested (Table 1). The severity of her infection resulted in the patient’s discharge with a PICC line to complete treatment with IV antibiotics, and subsequent close follow up with infectious disease specialists to ensure her remission.

As previously stated, recognized risk factors for pyelonephritis include frequent history of UTIs, maternal history of UTIs, changes or increase in sexual activity or practice or recent incontinence [10]. This patient presented with none of these risk factors. Furthermore, the likelihood of acute pyelonephritis as a result of infection with an XDR ESBL-producing *E. coli* is very rare, as these bacteria are seldom isolated even amongst hospitalized patients [12]. In fact, some studies have shown XDR isolates to be responsible for as few as 1.6% of nosocomial infections [4]. The resistance pattern of the *E. coli* isolated from this patient is unlike those previously observed in a population-based case–control study wherein 99% of *E. coli* isolates responsible for pyelonephritis in women were sensitive to ciprofloxacin, 91% were sensitive to ceftriaxone and 85% were sensitive to trimethoprim–sulfamethoxazole [10]. *E. coli* isolated from this patient were resistant to ciprofloxacin, ceftriaxone and trimethoprim–sulfamethoxazole. The patient additionally lacked criteria for complicated pyelonephritis, which is more likely to be caused by resistant bacteria, as she has a structurally and functionally normal genitourinary tract. Several studies have shown ESBL-producing *E. coli* UTIs in recently hospitalized patients, patients who have travelled, patients who have recently taken antibiotics, or patients on chronic steroid use or with diabetes mellitus however they have not been documented in young, otherwise healthy individuals without these risk factors [2,5,6,13].

Due to the significant resistance properties of the *E. coli* isolate responsible for this infection, as well as the lack of contact with healthcare or history of antibiotic use, consulting experts in infectious disease suggested the possibility of contact with raw meat at her job as the source of infection. The state food and safety administration report for her place of employment noted several violations around the time of her infection. These violations included lack of sanitizing stations and access to soap, use of wood boards to support meat trays, consecutive violations of pest infestation, and consecutive violations for unclean surfaces. It was also noted that the inspector “observed excess meat and debris buildup inside the cases and in the case tracks”, indicating some of these surfaces had come into contact with food products. While the inspector did not culture the workspace, they did suggest further inspection of the property and/or punitive action. Presently, the CDC does not consider food and water as possible sources of infection with ESBL-producing Enterobacteriaceae in the United States due to lack of data linking these workplaces with hospitalized infections [2]. Further research is certainly needed to determine if foodservice work is a possible source of infection for XDR ESBL-producing bacteria in the United States.

## 4. Conclusions

Community-acquired acute pyelonephritis is a common condition among women, including previously healthy and young women. While it accounts for almost 200,000 hospitalizations per year in the United States [11], the actual percentage of women diagnosed with this condition who require hospitalization is estimated to be around 7%, and most of these are either pregnant women, women with significant risk factors for the disease, or women older than 50 [10]. Despite our patient falling outside of these categories, and lacking any reported risk factors for resistant infection, she required an ICU stay and outpatient IV antibiotics due to the XDR *E. coli* infection. Without susceptibility studies on the urine culture and close observation, this patient may have had a very poor outcome. This case serves as an example that XDR and ESBL-producing *E. coli* strains can be present in patients without known risk factors and cause significant morbidity in otherwise healthy individuals. The successful treatment and overall positive outcome of this patient can provide guidance for the treatment of future patients presenting with infections caused by XDR ESBL-producing bacteria.

## Figures and Tables

**Table 1:** Susceptibility testing of the urine culture.

Antibiotic	Minimum Inhibitory Concentration (MIC)	Interpretation
Amoxicillin + Clavulanate	>32	Resistant
Ampicillin	>32	Resistant
Aztreonam	>64	Resistant
Cefalotin	>64	Resistant
Cefazolin	>64	Resistant
Cefepime	>64	Resistant
Cefpodoxime	>8	Resistant
Ceftriaxone	>64	Resistant
Cefuroxime	>64	Resistant
Ciprofloxacin	>4	Resistant
Extended-spectrum β-lactamase screen		Positive
Gentamicin	<1	Susceptible
Levofloxacin	>8	Resistant
Meropenem	<0.25	Susceptible
Nitrofurantoin	<16	Susceptible
Piperacillin + Tazobactam	64	Intermediate
Tetracycline	>16	Resistant
Tobramycin	>16	Resistant
Trimethoprim + Sulfamethoxazole	>320	Resistant

## Data Availability

Data sharing not applicable.
